# Factors related to cervical cancer and human papilloma virus awareness among rural women of southern Bangladesh: A cross-sectional study

**DOI:** 10.1016/j.gore.2024.101481

**Published:** 2024-08-15

**Authors:** Sattyajit Datta, Syed Baqui Billah, Anik Halder, Tarequr Rahman

**Affiliations:** a Medical Officer at Surgicare Diagnostic Center and Hospital, Bangladesh; b Department of Community Medicine at Sher-E-Bangla Medical College Hospital, Barishal, Bangladesh; c Indoor Medical Officer at Ibrahim General Hospital, Dhaka, Bangladesh; d Medical Officer at Evercare Hospital, Dhaka, Bangladesh

**Keywords:** Cervical cancer, Human papilloma virus, Knowledge, Attitude, Vaccine, Bangladesh

## Abstract

**Introduction:**

Cervical cancer is an important public health issue in Bangladesh. Despite government efforts, the awareness and healthcare utilization in rural areas are inadequate.

**Methodology:**

This cross-sectional study was conducted in six districts of Barishal division over 997 respondents aged 15–64 years. The demographic variables, accessibility to healthcare, vaccination status, awareness of HPV causing cervical cancer, and knowledge of the HPV vaccine were recorded through a structured questionnaire. T-test for univariate and chi-square test for bi-variate analysis were used. Logistic regression was used to determine factors associated with awareness.

**Results:**

Most of the participants were SSC passed (45.3%), unemployed (54.4%), and married (74.4%). While 83.5% were aware of cervical cancer, only 3.3% knew about HPV’s role. The majority (96.2%) had never been tested for HPV, albeit 79.5% knew about the vaccine, and only 2.45% were vaccinated. Knowledge levels were low, with only 1.4% demonstrating sound knowledge, mostly associating with higher income, residing closer to healthcare facilities, and being married. Bhola district participants had highest level of awareness, and the lowest was in Pirojpur. Education level was positively correlated with knowledge, with graduates being most aware. Logistic regression showed education, marital status, willingness to vaccinate daughters, and district were significantly related with better awareness, while income, distance, and age did not.

**Conclusion:**

Mass education and awareness campaigns may improve the awareness level in rural community.

## Highlights

• Human papilomma virus vaccination ratio is very low in southern Bangladesh.
• Education, marital status, willingness to vaccinate daughters were significantly related with better awareness.
• Good HPV awareness is less than 25% among study participants.
• Monthly income, age and distance from health centre does not influence HPV awareness.

## 1 Introduction

Cervical cancer is the fourth most common cancer among women worldwide, with approximately 70 % of cases involving infection with human papillomavirus (HPV) genotypes 16 and 18 (Hoque et al., 2021). Human Papilloma viruses (HPV) are small non-enveloped viruses, with a double-stranded circular DNA genome (Pinidis et al., 2016). In Asian geographic regions, the HPV 16/18-positive fraction accounted for almost 70 % of women with cervical cancer (Bao et al., 2008). There exists a gap in health-related information dissemination, even in developed nations (Chen et al., 2019). Regions facing limited access to healthcare and information, particularly rural areas of Bangladesh, bear a heightened risk due to low awareness levels. Despite governmental efforts to establish community clinics aimed at providing basic health services and raising awareness, merely one-third of women are aware of their existence, and a mere 15.92 % tend to utilize these clinics (Al-Zubayer et al., 2023, Yaya et al., 2017).

Bangladesh still contends with a 7.7 % prevalence of human papilloma virus infection (Nahar et al., 2014). One-third of the global burden of cervical cancer is shared among India, Sri Lanka, and Bangladesh combined (Sankaranarayanan et al., 2008). Considering that vaccination stands as the most effective preventive measure against HPV-induced cervical cancer, a leading cause of cancer-related deaths among Bangladeshi women, the urgency for intervention is evident (Bhuiyan et al., 2018). Women residing in rural areas are particularly at risk of HPV infection, with female sex workers facing a tenfold higher vulnerability (Peng et al., 2012). Despite advancements in medical knowledge and preventive measures, cervical cancer continues to afflict many women, especially those in underserved communities. Moreover, there has been an observable increase in hesitant attitudes toward vaccination since the 2009 influenza pandemic (Yaqub et al., 2014). This lack of awareness extends not only to rural, less-educated populations but also to university students in developed countries like Saudi Arabia (Aldawood et al., 2023).

While numerous studies have focused on urban areas of Bangladesh, there is a dearth of evaluations regarding rural regions (Bhuiyan et al., 2018, Chowdhury et al., 2022, Qayum and d O, Billah MM, Akhter R, Flora MS. , 2021, Sultana et al., 2023) as evidenced in the available literature. There has been adverse socioeconomic and demographic factors acting as barriers to vaccination acceptance (Guzman-Holst et al., 2020), we aim to exclusively target remote areas within the Barishal division of Bangladesh in this cross-sectional study. Our objectives are to assess the awareness toward HPV vaccination, analyzing the association of sociodemographic variables with the knowledge and attitude. This endeavor will bolster cervical cancer prevention, enabling the understanding of knowledge gaps, perceptions, and acceptance levels within this demographic. Ultimately, this comprehension is vital in devising targeted interventions and enhancing healthcare strategies tailored to meet the specific needs of these communities.

## 2 Methodology

We conducted this cross-sectional study among six districts of Barishal division over 997 willing respondents of 15–64 years of age with a pre-tested interview schedule from February to December 2023. We obtained the ethical approval prior to conducting the research from the Sher-E-Bangla Medical Ethics Committee. The study ensured compliance with ethical guidelines, including informed consent, confidentiality, and privacy of participant information throughout the data collection process. Written informed consent was obtained from each participant before their involvement in the study. A multi-stage cluster sampling method was used and our team conducted data collection at the residence of the participants. Participants were counseled about the process of answering each question by a doctor before filling out the questionnaire and they participated willfully and without any compensation. The interview schedule contained age, monthly income, district of residence, marital status, education level, occupation as demographic variables, distance from the nearest healthcare facility as an indicator of accessibility of healthcare, vaccination status, willingness to take the vaccine, willingness to vaccinate daughters as related variables, awareness that HPV causes cervical cancer, ever tested for HPV or not and knowledge of existing vaccine to assess HPV awareness and attitude. Data were collected through the administration of a structured questionnaire. Then data were entered into Microsoft excel where the preliminary cleaning of data was done. It was then exported to SPSS (v.23) where the second check of the data entry and quality was assessed. The scores of knowledge was determined by assessing the responses. The demographic and other factor variables were assessed with the knowledge variable. The quantitative variables were expressed as mean ± SD and the qualitative variables were presented as frequency and percentage. Five responses were selected to assess knowledge level, a) Do you know about cervical cancer? b) Do you know that HPV causes cervical cancer? c) Have you ever tested for HPV? d) Do you know that there is a vaccine to prevent the infection? e) Do you believe that vaccine is effective? Those who answered 4 or 5 questions positively were categorized as “Good knowledge”, those who answered 2 or 3 queries positively were marked as “some knowledge” and women who answered atlest 1 or no questions positively were categorized as “no knowledge”. Later, Those who portrayed at least one positive answer were categorized as “Aware” and those who did not were listed as “Not aware”. We used *t*-test for univariate and chi-square test for bi-variate analysis. The significant variables from the univariate analysis were taken into the model of logistic regression to determine the influence of the factors with the outcome variable (/knowledge). Secondary school certificate holders or less educated participants were kept as reference in education and Pirojpur district was taken as reference in district variable. The odds ratio (OR) from the logistic regression was accompanied by 95 % confidence interval (CI).

## 3 Results

The highest response was from Barishal district (n = 175,17.6 %), followed by 165(16.5 %) from Bhola,165(16.5 %) from Jhalakathi,165(16.5 %) from Patuakhali, 164(16.4 %) from Pirojpur and 163(16.3 %) from Barguna (not shown in table). Most of the participants (n = 452, 45.3 %) were secondary school certificate holders (10yearsofformaleducation), around a quarter of them were higher secondary certificate holders (12yearsofformaleducation) (n = 218, 21.9 %) and the rest 327of them were graduates (at least 16 years of formal education) (32.8 %). Five hundred and forty-two (54.4 %) women categorized them as unemployed, whereas 203 (20.4 %) women were employed and 252 (25.3 %) identified them as students. Two hundred and fifty-five (25.6 %) women were single and 742 (74.4 %) were married.832 (83.5 %) were aware of the disease called cervical cancer. However, only 33 (3.3 %) women knew that Human Papilloma virus causes cervical cancer. The number of women declared that they never tested for HPV was 959 (96.2 %) and 793 (79.5 %) women knew about the existence of vaccine, however, only 24 (2.45 %) of the total participants were vaccinated at that time. Four hundred and sixty-six (46.7 %) admitted that they believe vaccination is beneficial for prevention of the disease. Although 545 (54.7 %) were reluctant to take the vaccine in near future, 452 (45.3 %) stated that they will be vaccinated soon. One hundred and eleven (11.1 %) women denied to vaccinate their daughters despite learning about the merits of it, however, 886 (88.9 %) women were keen to get their daughters vaccinated. Only 1.4 % women demonstrated sound knowledge, 12.5 % accounted for some knowledge category and 86.1 % women demonstrated no to poor knowledge about the issue.

Only 24 (2.4 %) women were HPV vaccinated. The age was homogeneously distributed among the study participants as seen in Table 1 with other baseline variables. The participants with the knowledge of vaccine had nearly 2000 taka more income on average compared with those of no knowledge (p = 0.01), though with a very wide standard deviation of around 9000 taka/month. Those with at least some knowledge of the issue resided slightly closer to a healthcare facility. In terms of marital status, nearly 90 % married women demonstrated knowledge, approximately 10 % more than their unmarried counterparts. Awareness was the highest among participants hailing from Bhola, followed by Patuakhali and Barguna and lowest among those of Pirojpur. On the other hand, Women from Barishal and Jhalkathi district indicated nearly the same knowledge level percent wise. Knowledge level was positively correlated with the education level achieved, nearly 99 % among graduates, 90 % and 78 % among higher-secondary and secondary-school certificate holders respectively. About 84.5 % of the students surveyed, revealed awareness about the issue. Furthermore, 89.3 % of the unemployed women and 86.2 % of those who were employed declared the same.

**Table 1:** Sociodemographic factors related with HPV awareness.

**Variables**	**Not aware**	**Aware**	**Total**	**P**
**Age**	25.44 ± 5.54	24.58 ± 5.47		0.10
**Monthly Income(Tk)**	1864 ± 3904	3848 ± 8833		0.01
**Distance from the nearest health centre(km)**	3.66 ± 1.11	3.37 ± 2.27		0.16
**Marital Status**				<0.001
Single	50(19.6)	205(80.4)	255	
Married	75(10.1)	667(89.9)	742	
**District**				<0.001
Barishal	27 (15.4)	148 (84.6)	175	
Bhola	7 (4.2)	158 (95.8)	165	
Borguna	16 (9.8)	147 (90.2)	163	
Jhalkathi	26 (15.8)	139 (84.2)	165	
Patuakhali	13 (7.9)	152 (92.1)	165	
Pirojpur	36 (22.0)	128 (78.0)	164	
**Education**				<0.001
SSC	99(21.9)	353(78.1)	452	
HSC	22(10.1)	196(89.9)	218	
Graduate	4(1.2)	323(98.8)	327	
**Occupation**				0.14
Student	39(15.5)	213(84.5)	252	
Unemployed	58(10.7)	484(89.3)	542	
Employed	28(13.8)	175(86.2)	203	

Table 2 is describing vaccine related awareness. All vaccinated women demonstrated HPV awareness. About 87 % of the unvaccinated portion of the sample declared that they know about the issue. All of those who were interested to get the vaccine were well aware of the problem in question, whereas around three-fourths of the unwilling section illustrated awareness. In terms of vaccinating their female offspring, a paradoxical relation was observed. Those who declined to vaccinate their daughters were mostly aware of the disease (94.5 %), reflecting 8 % more aware population than their counterpart. Those who knew that the human papilloma virus causes cervical cancer and tested for it at least once in their lifetime showed better knowledge than those who did not. The attendants who knew the existence of an available vaccine showed better awareness (100 %) than those who did not (84.2 %).

**Table 2:** Knowledge and attitude.

**Variables**	**Not aware**	**Aware**	**Total**	**P**
**Vaccination Status**				0.06
Not vaccinated	125 (12.8)	848(87.2)	973	
Vaccinated	0(0.00)	24(1 0 0)	24	
**Will take vaccine**				
No	125(22.9)	420(77.1)	545	<0.001
Yes	0	452(1 0 0)	452	
**Vaccinate daughters**				
No	5(4.5)	106(94.5)	111	0.007
Yes	120(13.5)	766(86.5)	886	
**HPV causes C.C**				
No	125(12.9)	839(87.1)	964	0.03
Yes	0	33(1 0 0)	33	
**Ever tested for HPV**				
No	125(13)	834(87)	959	0.02
Yes	0	38(1 0 0)	38	
**Knowledge of vaccine**				
No	125(15.8)	668(84.2)	793	<0.001
Yes	0	204(1 0 0)	204	

We constructed a logistic regression model taking all the significant variables from the basic analysis **(**Table 3**).** Individuals with higher secondary levels of education were 2.4 times more likely to be HPV aware compared to those with lower education levels, whereas graduates were 21 times more aware than those with less education. Participants who reported to be married were about 3.53 times more likely to know about the issue compared to those who were single. Compared to participants from Pirojpur district, subjects from Bhola, Patuakhali, Borguna, Jhalkati and Barishal produced odds of 9.1, 4.7, 4.9, 2.2 and 2.4 times to be more aware respectively. Income, distance and age were not associated with awareness.

**Table 3:** Logistic regression of factors related to awareness of HPV.

**Variables**	**OR(95 %CI)**	**P**
**Education**		<0.001
SSC or below	Ref.	
HSC	2.4 (1.3–4.4)	0.006
Graduate	21.1(6.8–65.8)	<0.001
**Income**	1.00 (1.00–1.00)	0.16
**District**		<0.001
Pirojpur	Ref.	0.01
Bhola	9.1(3.5–23.3)	<0.001
Patuakhali	4.7(2.1–10.3)	<0.001
Borguna	4.9(2.2–10.9)	<0.001
Jhalkati	2.2(1–4.4)	0.03
Barishal	2.4(1.2–5)	0.02
**Married**	3.53 (2.73–7.65)	<0.001
**Will vaccinate daughter**	0.29 (0.09–0.70)	0.01
**Distance**	0.99 (0.94–1.20)	0.27
**Age**	0.96 (0.95–1.02)	0.58

## 4 Discussion

The findings highlight shortcomings in awareness levels regarding cervical cancer, Human Papillomavirus (HPV), and vaccination among rural women of Barishal division and factors affecting it. Health literacy plays an important role in reproductive knowledge and may impact willingness to be vaccinated. (Joshi et al., 2020, Kilfoyle et al., 2016) In low- and middle-income countries, insufficient knowledge and perceptions hinder overall vaccine willingness, while superstitions and fear account for a vast majority of unvaccinated individuals. Challenges such as high costs, logistical complexities, vaccine safety concerns, socio-cultural factors contribute to the limited uptake of vaccines in these regions. (Bari et al., 2021, Brannen et al., 2023, Toh et al., 2017) Vaccination campaigns and government –funded programs often hindered by hesitancy and lack of awareness among population. (Fayyaz and Satti, 2022, Nguyen, n.d).

Knowledge gap exists between urban and rural women, encompassing crucial areas such as family planning, care during pregnancy, safe motherhood, newborn care, birth spacing, and a general lack of awareness about cervical cancer among the population. (Haque et al., 2015).

The association between education and awareness was evident, with higher levels of education correlating positively with increased knowledge about cervical cancer, HPV, and vaccination. Graduates displayed the highest awareness (99 %), followed by individuals with a higher secondary certificate (90 %), and those with a secondary school certificate (78 %). This reinforces the importance of education in disseminating information about preventive healthcare measures. Marital status also played a significant role, as nearly 90 % of married women exhibited awareness, which was approximately 10 % higher than unmarried participants. This trend suggests that married individuals might have better access to health-related information or increased exposure to healthcare conversations within their households. Geographic location, particularly district-wise variations, demonstrated noteworthy discrepancies in awareness levels. Bhola district exhibited the highest awareness, while Pirojpur displayed the lowest. While the reason for this discrepancy demands further research, it is important to note that the higher number of both national and international NGO operations in the Bhola district could be one of the reasons. The study identified a correlation between vaccination status and knowledge about cervical cancer. All vaccinated participants displayed awareness about the disease and HPV, emphasizing the impact of vaccination programs in imparting knowledge. HPV testing is the primary cervical cancer screening modality in Bangladesh. However, a substantial proportion (96.2 %) of respondents had never undergone HPV testing, despite the test being available in every sub-district health complex, medical colleges and tertiary care hospitals, signifying a significant gap in preventative healthcare practices. The HPV screening test is done at free of cost in government health facilities including sub-district health complexes, medical colleges and tertiary care hospitals. The lack of knowledge stands out as a pivotal factor contributing to the escalating incidence of cervical cancer in Bangladesh. Concurrently, low- and middle-income countries (LMICs) face a challenge characterized by the co-infection of human immunodeficiency virus (HIV) and Human Papillomavirus (HPV). This dual infection amplifies the risk of cervical cancer and identifies a specific population with an urgent and critical need for effective cervical cancer prevention strategies. (Alam et al., 2022, Sahasrabuddhe et al., 2012).

Studies conducted in various countries revealed alarmingly low awareness levels regarding Human Papillomavirus (HPV) and the HPV vaccine. In Malaysia, only 11.6 % were aware of HPV, with a mere 7.8 % having knowledge of the newly released HPV vaccine (Wong, 2011). Similarly, a Nepalese study reported an overall awareness of HPV among women at 15.4 % (Johnson et al., 2014). In India, a study highlighted an overall low frequency of awareness, standing at 15 %, concerning HPV and cervical cancer (Hussain et al., 2014, Sabeena et al., 2015). These findings collectively underscore the need for widespread education and awareness campaigns to address the knowledge gap (Johnson et al., 2014, Hussain et al., 2014, Sabeena et al., 2015). There are numerous precedents of this effort in other arenas of Bangladesh health sector, for instance, the extended program on immunization (EPI), vitamin A capsule program and ante-natal checkup program.

Educational initiatives aimed at increasing awareness should focus on regions with lower awareness levels, tailoring information to address specific demographic needs, for example, communicating in local language, eliminating cultural barriers and superstitions, using pictures and illustrations to educate the target population. As we can see that most of the 111 women who declined vaccination for their daughters were well aware of the HPV and its association with the cervical cancer, the question arises naturally, why they are acting so? By exploring local legends, we learned that this phenomenon is nothing new. This hesitancy is present since the very first vaccine came into existence. The COVID-19 pandemic further complicated the situation. The answer lies within their superstitions and phobias. Similar to the different countries worldwide, some Bangladeshi people also have phobias and superstitions regarding the safety and efficacy of vaccines (Bagheri Sheykhangafshe and Esmaeilinasab, 2022). While this study explores the attitude of Bangladeshi women towards vaccination, the attitude of Bangladeshi men should also be addressed because health decisions in Bangladeshi families is made by men and wider family unit most of the time. Policymakers should consider incorporating comprehensive cervical cancer education into school curricula and implementing accessible vaccination programs to increase uptake among the population, especially among underserved communities.

## 5 Funding statement

This research was conducted without any financial support from external sources.

## Author contribution

Professor Syed Baqui Billah conducted statistical analyses involved in the study.

Sattyajit Datta was involved in data collection, data cleaning, statistical analyses, writing of methodology and results section.

Md Tarequr Rahman was involved in data collection, writing of introduction section and referencing.

Anik Halder was involved in data collection, writing of discussion section and referencing.

## Declaration of Generative AI and AI-assisted technologies in the writing process

During the preparation of this work the authors used GPT 4 in order to streamline the writing. After using this service, the authors reviewed and edited the content as needed and take full responsibility for the content of the publication.

## CRediT authorship contribution statement

**Sattyajit Datta:** Writing – review & editing, Writing – original draft, Visualization, Validation, Supervision, Software, Resources, Project administration, Methodology, Formal analysis, Data curation, Conceptualization. **Syed Baqui Billah:** Writing – review & editing, Supervision, Resources, Methodology, Formal analysis, Conceptualization. **Anik Halder:** Writing – review & editing, Writing – original draft. **Tarequr Rahman:** Writing – review & editing, Writing – original draft.

## Declaration of Competing Interest

The authors declare that they have no known competing financial interests or personal relationships that could have appeared to influence the work reported in this paper.
